# Synthesis of Human Bone Morphogenetic Protein-2 (hBMP-2) in *E. coli* Periplasmic Space: Its Characterization and Preclinical Testing

**DOI:** 10.3390/cells10123525

**Published:** 2021-12-14

**Authors:** João E. Oliveira, Miriam F. Suzuki, Renata Damiani, Eliana R. Lima, Kleicy C. Amaral, Anderson M. S. Santos, Geraldo S. Magalhães, Leonardo P. Faverani, Luís A. V. D. Pereira, Paolo Bartolini

**Affiliations:** 1 Instituto de Pesquisas Energéticas e Nucleares, IPEN–CNEN, Av. Prof. Lineu Prestes 2242, São Paulo 05508-000, SP, Brazil; jeolivei@ipen.br (J.E.O.); mfsuzuki@ipen.br (M.F.S.); kleicy12@hotmail.com (K.C.A.); 2 Biosintesis P & D, São Paulo 05508-000, SP, Brazil; redamiani@hotmail.com (R.D.); lima-eliana@hotmail.com (E.R.L.); 3 Department of Diagnosis and Surgery, School of Dentistry, Sao Paulo State University, UNESP, Araçatuba 16015-050, SP, Brazil; andersonmaikon@hotmail.com (A.M.S.S.); leonardo.faverani@unesp.br (L.P.F.); 4 Immunopathology Laboratory, Instituto Butantan, São Paulo 05503-900, SP, Brazil; Geraldo.magalhaes@butantan.gov.br; 5 Department of Biochemistry and Tissue Biology, Institute of Biology, State University of Campinas, UNICAMP, Campinas 13083-970, SP, Brazil; lviolin@unicamp.br

**Keywords:** hBMP-2, periplasmic expression, calvarial critical-size defect, osteoinductor

## Abstract

Human BMP-2, a homodimeric protein that belongs to the TGF- β family, is a recognized osteoinductor due to its capacity of inducing bone regeneration and ectopic bone formation. The administration of its recombinant form is an alternative to autologous bone grafting. A variety of *E. coli-*derived hBMP-2 has been synthesized through refolding of cytoplasmic inclusion bodies. The present work reports the synthesis, purification, and characterization of periplasmic hBMP-2, obtained directly in its correctly folded and authentic form, i.e., without the initial methionine typical of the cytoplasmic product that can induce undesired immunoreactivity. A bacterial expression vector was constructed including the DsbA signal peptide and the cDNA of hBMP-2. The periplasmic fluid was extracted by osmotic shock and analyzed via SDS-PAGE, Western blotting, and reversed-phase high-performance liquid chromatography (RP-HPLC). The purification was carried out by heparin affinity chromatography, followed by high-performance size-exclusion chromatography (HPSEC). HPSEC was used for qualitative and quantitative analysis of the final product, which showed >95% purity. The classical in vitro bioassay based on the induction of alkaline phosphatase activity in myoblastic murine C2C12 cells and the in vivo bioassay consisting of treating calvarial critical-size defects in rats confirmed its bioactivity, which matched the analogous literature data for hBMP-2.

## 1. Introduction

Human bone morphogenetic protein-2 (hBMP-2) was first discovered when Urist et al. established the biological basis of morphogenesis [1,2,3]. Its purification from demineralized bone matrix and definitive characterization were first performed by Reddi et al. [4,5,6].

hBMP-2 is a homodimeric cysteine-knot protein that belongs to the transforming growth factor-β (TGF-β) family, whose structure is stabilized through dimerization and an additional intermolecular disulfide bond [7,8]. It was soon observed that hBMP-2 is one of the most efficient osteoinductors ever described, especially because of its capacity of inducing bone regeneration and ectopic bone formation in adult vertebrates. Its cloning and synthesis in CHO cells were first carried out at the Genetic Institute of Cambridge, MA, USA [9,10], administration of the recombinant form being an alternative to autologous bone grafting, used in a variety of orthopedic and dental applications, such as spinal fusions and oral surgery, and in the repair of bone, cartilage, tendons, and ligaments [11,12,13,14,15]. In particular, it has been demonstrated that the application of recombinant hBMP-2, together with an absorbable collagen sponge (ACS), induces new bone formation in mandibular and cleft palate defects, whose results have been compared to those of autogenous bone grafts [16,17].

One of the most important and widely used recombinant preparation of hBMP-2 is Infuse^®^ from Medtronic (Minneapolis, MN, USA), obtained from CHO cells and even considered to be a type of international reference preparation for clinical use [18,19]. A variety of *E. coli*-derived preparations obtained through solubilization and refolding of cytoplasmic inclusion bodies also demonstrated good biological activity [8,13,20,21,22,23]. Different in vitro and in vivo studies also proved the comparable osteoinductive capacity and clinical efficacy of these preparations [24,25,26,27].

In the present work, the synthesis of hBMP-2 was achieved in the periplasmic space of genetically modified *E. coli*, which provides the naturally refolded protein in its authentic form.

After osmotic shock, the periplasmic fluid was extensively purified via three chromatographic steps, followed by a physical-chemical and immunological characterization via RP-HPLC, HPSEC, SDS-PAGE and Western blotting, in comparison with the well-known cytoplasmic Met-hBMP-2 from GenScript (Piscataway, NJ, USA). The classical in vitro and in vivo bioassays, based respectively on alkaline phosphatase induction in myoblastic murine cells C2C12 and on new bone formation in calvarial critical-size defects in rats, confirmed the osteoinductive properties of our hBMP-2.

A preliminary pilot-scale production of this hBMP-2 was also carried out under well-controlled bioreactor conditions, showing the real possibility of obtaining this protein in useful amounts.

## 2. Materials and Methods

### 2.1. Construction and Testing of the Expression Vector

**Figure 1:**
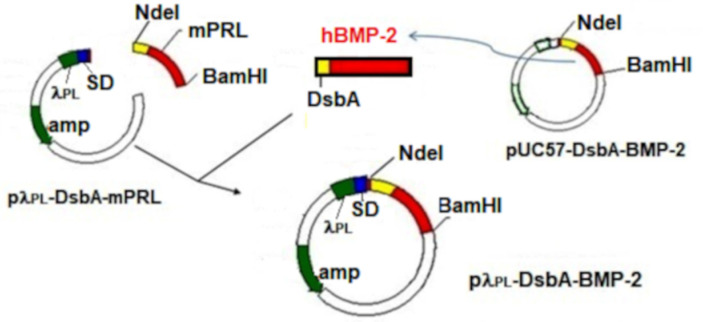
Construction scheme of the bacterial expression vector pλ_PL_-DsbA-BMP-2 for the periplasmic expression of authentic hBMP-2.

The pUC57-derived plasmid containing the restriction sites EcoRI, NdeI, BamHI, the bacterial signal sequence of DsbA (disulfide bond formation protein A), and hBMP-2 cDNA (NCBI Reference Sequence: AF040249.1—*Homo sapiens* bone morphogenetic protein-2 gene, complete cds), was commercially synthesized by GenScript (Piscataway, NJ, USA) providing the plasmid pUC57-DsbA-BMP-2 (Figure 1).

The fragment DsbA-hBMP-2 was withdrawn from this plasmid by NdeI and BamHI digestion and purified via agarose gel (1%) electrophoresis in TAE (Tris-acetate EDTA) buffer. Another fragment including the λ_PL_ promoter, the Shine–Dalgarno (SD) sequence, and the gene for ampicillin resistance (Amp^R^) was obtained via NdeI, BamHI digestion from the pλ_PL_-DsbA-mPRL expression vector, previously constructed by the same research group [28]. The ligation reaction between the two fragments was carried out using T4 DNA ligase (New England Biolabs Inc., Ipswich, MA, USA), incubating overnight at 4 °C. The molar ratio between the first fragment (insert) and the second fragment (vector) was 5:1. The correct construction of the final expression vector pλ_PL_-DsbA-BMP-2 (2966 bp) was checked in 1% agarose gel, by BamHI and NdeI digestion, providing the two expected fragments of 2559 bp and 407 bp. After transforming competent bacteria (DH5α) with the ligation product, 10 colonies were obtained, which were tested by PCR for the presence of pλ_PL_-DsbA-BMP-2, using the following two primers: sense GGCGCATATGAAAAAGATTTGGCTGG and antisense ATTAGGATCCTACTAGCGACACCCAC. Two colonies were found to be positive, the PCR product being sequenced at the Human Genome and Stem Cell Research Center (HUG-CELL) of the Institute of Biosciences of the University of São Paulo (São Paulo, Brazil), confirming the correct sequence in comparison with pUC57-hBMP-2 and with the theoretical hBMP-2 sequence, utilizing the BioEdit Sequence Alignment Editor Software [29].

### 2.2. Heparin Affinity and HPSEC Purification

The W3110 *E. coli* strain, transformed with pλP_L_-DsbA-BMP-2 and containing the thermosensitive repressor of transcription cIts, was grown in Erlenmeyer flasks, and the periplasmic fluid was extracted by osmotic shock, as previously described [30]. The first hBMP-2 purification step was carried out on this periplasmic fluid by heparin affinity chromatography, at room temperature, introducing significant modifications in the method described by Vallejo et al. [13]. As an example, the concentrated periplasmic fluid (~80 mL), containing ~1.6 mg hBMP-2, was extensively dialyzed against 100 mM Tris-HCl (pH 7.5), overnight, using a membrane SnakeSkin Dialysis Tubing (Thermo Scientific, Rockford, IL, USA), 22 mm dry I.D. with a 3.5 kDa cut off, and passed through a 0.45 µm-pore-size filter, hydrophilic PVDF, 47 mm-diameter membrane GV Durapore^®^ (Merck Millipore, Cork, Ireland). The dialyzed fluid was applied to a 5 mL HiTrap Heparin HP column (GE Healthcare Bio-Science AB, Uppsala, Sweden), which had been equilibrated with 5 column volumes (CV) of dialysis buffer. After washing the column with 5 CV of the same dialysis buffer, the monomeric and dimeric forms of hBMP-2 were eluted via a 0 to 0.3 M NaCl gradient in a 50 mL elution volume. The eluted fractions containing dimeric hBMP-2 (~3 mL) were applied (6 fractions of 500 µL) to preparative HPSEC, obtaining finally a total of 574 μg of purified hBMP-2 in 2 mL (total yield = 36%), which was stored at −70 °C.

### 2.3. SDS-PAGE and Western Blotting

Periplasmic hBMP-2 and samples from the purification steps were analyzed by 15% polyacrylamide gel electrophoresis (SDS-PAGE) under non-reducing conditions, as previously described, Coomassie Brilliant Blue G-250 being used for the staining [31]. For Western blotting, the semi-dry transfer technique on nitrocellulose membrane was used, with anti-hBMP-2 affinity-purified rabbit IgG, 1:2000 (Biovision, Milpitas, CA, USA) and goat anti-rabbit IgG conjugated to horseradish peroxidase (1:5000). Proteins were visualized with Luminata Forte (Merck, Burlington, MA, USA) on CL-Xposure ^TM^ Film (Thermo Scientific, Rockford, IL, USA).

### 2.4. Analytical Reversed-Phase High-Performance Liquid Chromatography (RP-HPLC)

RP-HPLC was performed on a Jupiter C4 column (Phenomenex, Torrance, CA, USA), 250 mm × 4.6 mm I.D., 5 µm particle size, and 300 Å pore size, connected to a guard column cartridge (4 mm × 3 mm I.D., 3 µm particle size) in a Shimadzu model SCL-10A apparatus. Elution was carried out at 30 °C with UV detection at a wavelength of 220 nm. For the gradient, two solutions were used: solution A, TFA 1:1000 in H_2_O, and solution B, 10% A in acetonitrile. The linear gradient used for hBMP-2 elution went from 30% B (*v*/*v*) to 60% B (*v*/*v*) over 30 min, followed by an isocratic step with 60% B for 5 min.

### 2.5. High-Performance Size-Exclusion Chromatography (HPSEC)

HPSEC was used for analytical and preparative purposes, employing a G2000 SW column (600 mm × 7.5 mm I.D., particle size of 10 μm and pore size of 125 Å from Tosoh Bioscience (Montgomeryville, PA, USA), in a Shimadzu model SCL-10 A apparatus. UV absorbance detection was at 220 nm, with a flow rate of 1.0 mL/min, using 0.15 M NaCl in 0.02 M sodium phosphate buffer, pH 7.0, as the mobile phase for isocratic elution.

### 2.6. In Vitro hBMP-2 Bioassay in C2C12 Cells

The in vitro biological activity of hBMP-2 was determined by measuring the induction of alkaline phosphatase activity in murine myoblastic C2C12 cells [32]. Briefly, C2C12 cells (from ATCC^®^-CRL-1722) were grown in DMEM medium with 2 mM L-glutamine, 0.1 mM non-essential amino acids, 1 mM sodium pyruvate, and 10% fetal bovine serum at 37 °C and 5% CO_2_. One hundred microliters of C2C12 cells (3 × 10^5^ cells/mL) was added to a 96-well plate, and the medium was replaced after 24 h with fresh medium, 2% calf serum, and different concentrations of hBMP-2, each concentration being analyzed in duplicate. After 72 h, the cells were lysed in 0.2 mL of buffer A (0.1 M glycerol, pH 9.6, 1% NP-40, 1 mM MgCl_2_, and 1 mM ZnCl_2_). Then, 50 µL of cell lysates was mixed with 150 µL 0.3 mM p-nitrophenyl-phosphate (Sigma-Aldrich, Saint Louis, MO, USA) in buffer A, incubating at 37 °C for 30 min. Alkaline phosphatase activity was determined by reading the absorbance at 405 nm with a Multiskan EX Microplate Reader (Thermo Electron Corporation, Beverly, MA, USA).

### 2.7. Large-Scale Fermentation under Controlled Bioreactor Conditions

Fed-batch bioreactor experiments were carried out in a 20 L Laboratory Bioreactor (New MBR, Zurich, Switzerland), adding 100 μg/mL of ampicillin [30]. The bioreactor operated at pH 7.2 and dissolved O_2_ was maintained above 20% of air saturation by increasing the agitation speed from 200 to 900 rpm and adding 1 volume of air per volume of medium per minute (VVM), to avoid O_2_ limitation. A complex culture medium, a two- or four-fold concentrate of the HKSII medium reported by Jensen and Carlsen [33], was used. For inoculation, 10% of the initial total volume (i.e., 500 mL) of LB medium containing the transformed W3110 strain that had reached 0.5 A_600_ units was used, the culture being maintained at 30 °C until activation. The carbon source (glucose in this case) addition was started at a biomass concentration corresponding to 5 A_600_ units, the feed rate being adjusted to the desired value and kept constant until the end of the cultivation. Activation at 42 °C started when A_600_ approached a plateau. The glucose feeding rates, when not specified differently, were 3.6 gL^−1^h^−1^ and 5.4 gL^−1^h^−1^ during the activation step.

### 2.8. In Vivo Bioassay for the Determination of the Osteoinductive Potential by Treating Calvarial Critical-Size Defects in Rats

The in vivo study was approved by the local ethics committee for the use of animals (# 00285-2019) and followed the guidelines for animal research [34]. Twelve male rats (*Rattus novergicus albinus*, Wistar), six months old, weighing between 250 and 300 g, were submitted to a bilateral critical-size defect in the calvarias. The rats were kept in a cage (3 per cage), in an environment with a controlled cycle of light (12 h light–dark), controlled temperature (22 ± 2 °C), and food and water ad libitum, except in the preoperative 8 h, when they underwent fasting.

The animals were randomly divided into four groups, according to the material used to cover the defect. i.e., NC Group: just blood clot (negative control); ACS Group: absorbable collagen sponge alone; INF Group: ACS with commercial rhBMP-2 (Infuse^®^ Bone Graft—Medtronic, Memphis, TN, USA); hBMP-2 Biosintesis Group: collagen membrane with our bacterial hBMP-2.

The amount of added hBMP-2 was 2.5 μg/defect for the Infuse^®^ and 13.5 μg/defect for the hBMP-2 Biosintesis groups, a difference that approximately reflected the difference in the in vitro bioactivities between Infuse^®^ and hBMP-2 GenScript found in a previous work [31].

The sample size was determined via the software SigmaPlot 12.0 (Exakt Graphs and Data Analysis, San Jose, California, USA), based on data for newly formed bone area from the study by Nakamura et al. [35], which showed a mean difference of 1.77 and a standard deviation of 0.57, with an alpha of 0.05. Accordingly, five defects per group would be enough to obtain a power test of 95%, but, since a bilateral defect was adopted, six defects per group were performed.

#### 2.8.1. Surgical Procedure

After fasting for 8 h, the animals were sedated with ketamine (50 mg/kg) and xylazine (5 mg/kg) intramuscularly and received local anesthesia (Mepiadre100^®^, DFL, Rio de Janeiro, RJ, Brazil) in the area to be operated. Trichotomy and antisepsis (Degerming and Topic Polyvinyl Pyrrolidone Iodine) were performed on the calvaria region. A “V” access, with the apex in the anterior region of the calvaria, was made to expose the parietal region, followed by osteotomy on both parietal bones with a trephine, creating a critical-size defect of five millimeters in diameter. According to the groups (ACS, INF, and hBMP-2 Biosintesis), the defects were covered or not with any material, with only the blood clot remaining in the negative control (NC). The defects were finally covered by the flap and sutured with Nylon 5.0 (Mononylon, Ethicon, Johnson Prod., São José dos Campos, SP, Brazil). All animals received a single dose of antibiotic intramuscularly (0.2 mg/kg; Pentabiotic—Zoetis^®^, Bauru, SP, Brazil).

At 60 days after surgery, all animals were euthanized with an overdose of sodium thiopental 150 mg/kg, intraperitoneally, and the calvarias was collected and stored in 10% formaldehyde for 48 h, followed by histological and histometric analysis.

#### 2.8.2. Histological and Histometric Analyses

After 48 h in formalin solution, the samples were washed for 12 h in water and decalcified during 8 weeks in ethylenediaminetetraacetic acid. The samples were dehydrated in alcohol (70–100%), diaphanized in xylol, and embedded in paraffin. Five micron longitudinal tissue sections were obtained. Hematoxylin and eosin staining was used for histological assessment. The images were obtained on an optical microscope (Leica Microsystems, Heidelberg, Germany) from the central area of the bone defect. The histometric analysis was performed by an independent and blinded examiner (A.M.S.S.) using the Image J, version 1.53f 25; Software for Image Processing and Image Analysis, NIH, USA, 2020. The Newly formed Bone Area (NBA—µm^2^) was measured. The analysis was performed at two different times (at least 15 days between each analysis) by the same examiner. The Kappa test showed a high level of agreement (k = 0.90).

#### 2.8.3. Statistical Analysis

The quantitative data for NBF were analyzed to test homogeneity through the Shapiro–Wilk test. The non-parametric one-way Kruskal–Wallis test followed by the Student–Newman–Keuls post-test was applied with a level of significance of 5% in SigmaPlot 12.0 software (Exakt Graphs and Data Analysis, San Jose, CA, USA).

## 3. Results

### 3.1. Expression of hBMP-2 in the Periplasm

**Figure 2:**
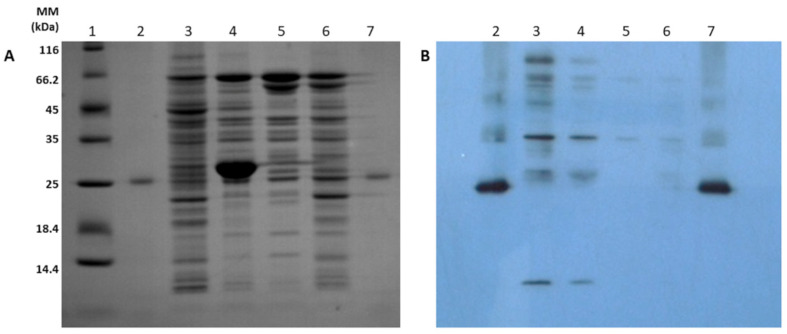
(**A**) SDS-PAGE analysis of periplasmic fluid obtained with the W3110 strain + repressor gene at 30 °C and 42 °C. (1) Molecular marker, (2) met-hBMP-2 standard from GenScript, (3) W3110 periplasmic fluid at 42 °C, (4) W3110 periplasmic fluid at 30 °C, (5) W3110 negative control at 30 °C, (6) W3110 negative control at 42 °C, (7) met-hBMP-2 standard from GenScript. (**B**) Western blot analysis obtained under the same conditions described in (**A**).

After testing the correct sequence of the pλ_PL_-DsbA-hBMP-2 expression vector and the size and presence of the different components, the main objective was to prove hBMP-2 expression in the periplasm of the W3110 strain. Figure 2A shows the SDS-PAGE analysis of the periplasmic fluid, after cultivation at 30 °C and 42 °C of the transformed and untransformed (negative control) W3110 strain. It is important to note that the presence of the temperature-sensitive repressor (cIts) should potentially stop hBMP-2 synthesis at 30 °C, favoring bacterial growth, then allowing its maximum expression at 42 °C, with repressor deactivation. Unfortunately, this approach proved to be inconclusive due to the presence of numerous protein bands of difficult interpretation. The only clear result was related to the sole presence of the dimeric form of the reference preparation of met-hBMP-2 (~26 kDa) in wells 2 and 7. Protein bands close to this position were observed in all samples, including the negative control.

In contrast, the corresponding Western blot in Figure 2B provided much more information. Thus, the reference preparation from GenScript (wells 2 and 7) was clearly mainly dimeric, but it showed at least three polymeric forms and absolutely no monomer. The dimeric form of our hBMP-2 appeared a little above the reference and was about 2 times more intense at 42 °C. However, there were numerous polymeric or aggregated forms, in the regions of 35–38 kDa and 60–80 kDa, all of which were more intense at 42 °C, together with the clear and unexpected presence of the monomer (~13 kDa). We therefore concluded that the repressor was indeed working, but not very efficiently. The fact that the dimeric form of hBMP-2 migrated a little above the dimeric form of met-hBMP-2 might be due to a structural and conformational difference caused by the absence of the initial methionine. Concerning the implementation of this assay, it is of interest to emphasize that it was quite difficult to find a good working anti-hBMP-2 antibody. Good antigen–antibody reaction after Western blotting was only obtained thanks to an affinity-purified anti-hBMP-2 rabbit IgG (1:2000) from Biovision.

**Figure 3:**
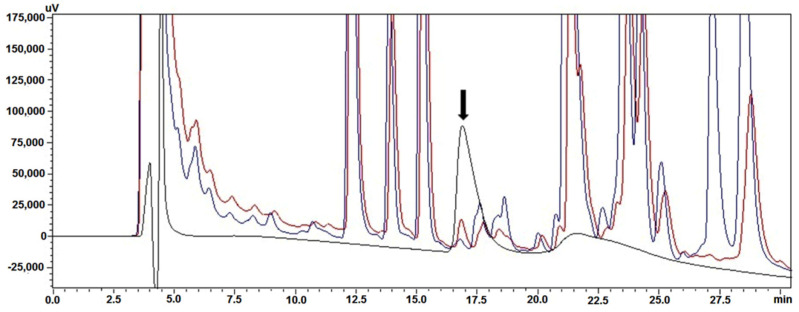
RP-HPLC analysis comparing hBMP-2 GenScript (black chromatographic trace) with the results using the repressor at 30 °C (blue trace) and at 42 °C (red trace). The arrow indicates the position of hBMP-2.

For completeness, it was deemed necessary to confirm the presence of hBMP-2 in W3110 periplasm by employing a different technique. This was done by using RP-HPLC, a chromatographic technique in which the retention time (t_R_) of a given protein depends directly on its hydrophobicity. Figure 3 shows the superimposition of three RP-HPLC chromatograms, run on the same day (“super compare”). The black chromatographic trace corresponds to the reference preparation, which defines exactly the position of hBMP-2 (t_R_ = 17.4 min), while the two chromatographic traces below correspond to the W3110 strain, harboring the vector λ_PL_-DsbA-BMP-2, activated at 42 °C (red trace) and 30 °C (blue trace), respectively. The identity of our periplasmic hBMP-2 was thus confirmed, and again, the peak area obtained at 42 °C was approximately 2–3 times larger than that obtained at 30 °C.

**Figure 4:**
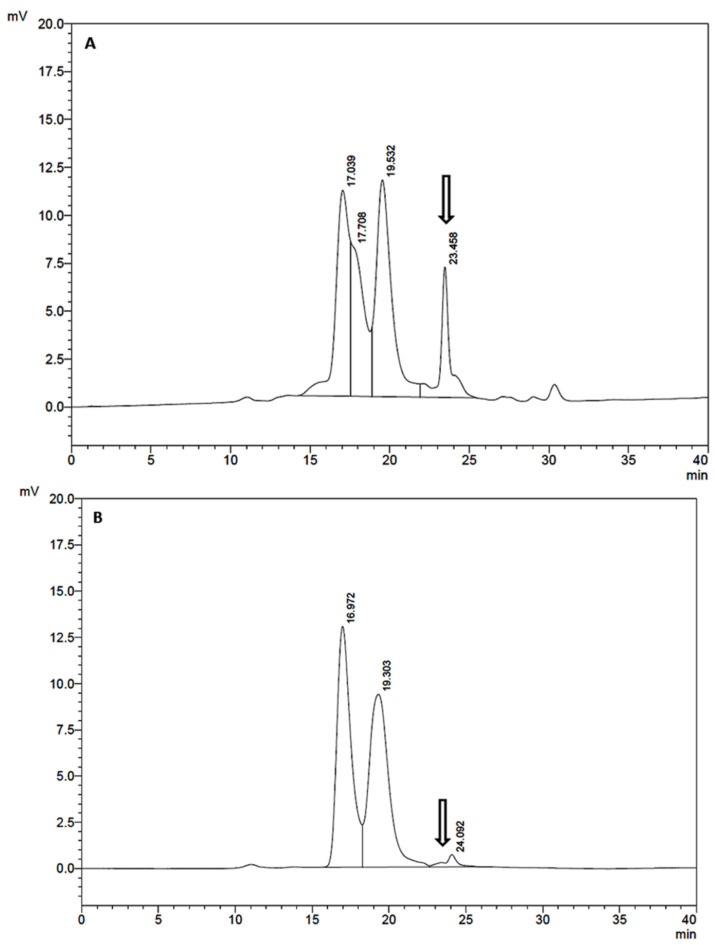
HPSEC analysis comparing the heparin affinity-eluted product obtained with the W3110 pλ_PL_-DsbA-BMP-2-producing strain (**A**) and the W3110 negative control (**B**). The arrow indicates the position of hBMP-2.

A similar comparison was also carried out via HPSEC after the first purification step on a heparin column, considering that this type of chromatography, based on molecular mass, normally represents the best quantifying technique. Figure 4A presents the chromatogram of our partially purified product, while Figure 4B shows the chromatogram obtained with the product derived from the untransformed W3110 strain (negative control) and submitted to the same purification process, which obviously lacks the hBMP-2 peak. On the same day, the reference preparation from GenScript presented t_R_ = 23.24 min.

**Figure 5:**
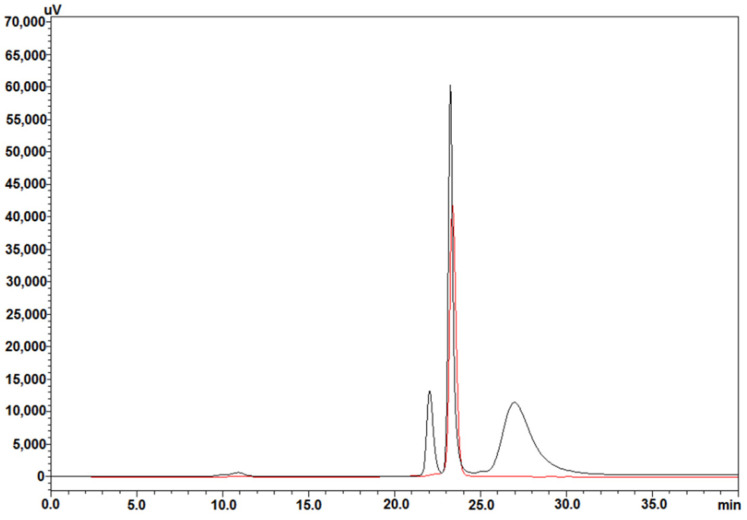
HPSEC analysis comparing hBMP-2 Biosintesis (red trace) after three purification steps (HiTrap Heparin HP, HPSEC-1, and HPSEC-2) with the hBMP-2 reference preparation from GenScript (black trace).

The second purification step was based on the same HPSEC, run in preparative mode, and the chromatogram of the obtained product is shown in Figure 5, where the black trace is purified hBMP-2, and the red trace corresponds to the reference preparation. Again, the t_R_ were remarkably similar: 23.36 min and 23.24 min for hBMP-2 Biosintesis and hBMP-2 GenScript, respectively. This HPSEC-eluted product, that had undergone three purification steps (Heparin-affinity + HPSEC-1 + HPSEC-2), was considered to be the final product.

Table 1 reports the results of the purification processes up to the first HPSEC. Total protein (μg/mL) was determined via the classical assay based on the bicinchoninic acid assay (BCA); in the case of met-BMP-2 GenScript, the 5 μg declared value was based on the nominal value. This preparation is in fact provided in vials with the following declaration: “it contains 1 mg lyophilized product, with >95% purity, in dimeric form (~26 kDa) and biologically active for its capacity to induce alkaline phosphatase in C2C12 cells”. Some recommendations concerning the poor stability of the product are also provided [31]. The quantity of hBMP-2 (μg/mL) was determined by HPSEC versus a standard protein repeatedly quantified on this column, and the specific activity was calculated as absorbance per microgram (A_405_/μg), after determining the biological activity in C2C12 cells. The latter parameter provided a potency of 98% for our product in comparison with the reference preparation.

### 3.2. In Vitro Bioassay in C2C12 Cells

**Figure 6:**
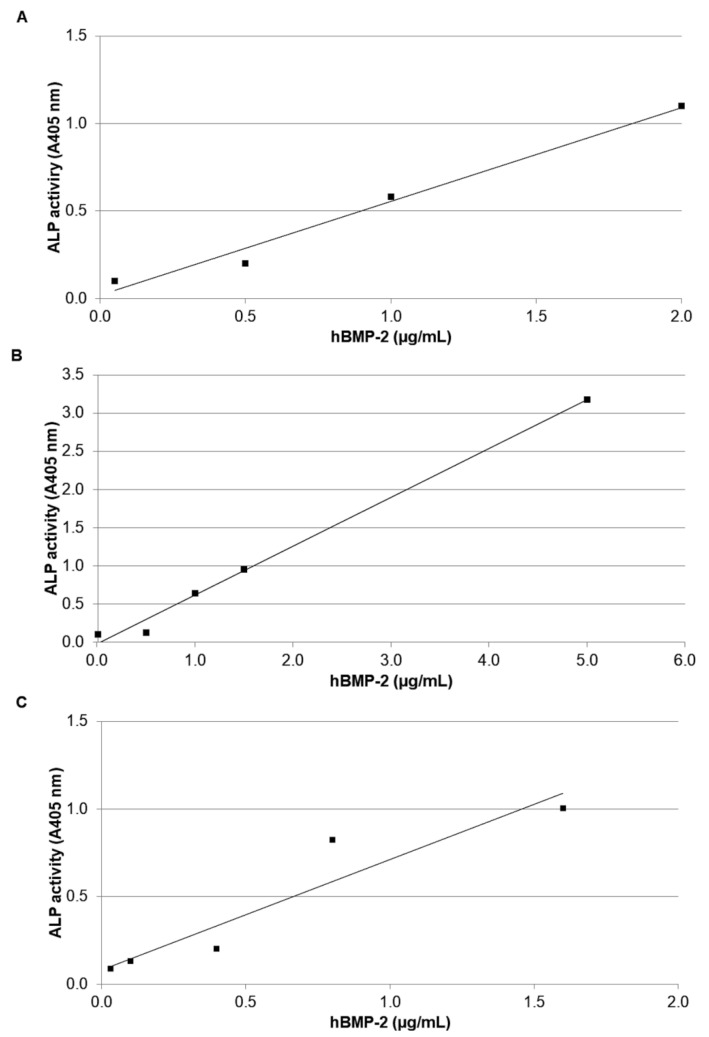
In vitro biological activity determined by alkaline phosphatase induction in C2C12 cells. (**A**) Data adapted from [13]; (**B**) standard met-hBMP-2, GenScript; (**C**) purified hBMP-2 Biosintesis.

An inter-preparation, inter-laboratory comparison was also performed, based on the biological assay in C2C12 cells. As we can see in Figure 6, three graphs are presented. The first (Figure 6A) is adapted from [13], analyzing their renatured and purified cytoplasmic product, the second (Figure 6B) represents our analysis carried out on the GenScript product, while the third (Figure 6C) represents our analysis carried out on our purified product. The related dose–response equations are the following:

(A) Y = 0.533X + 0.024 (n =4; r = 0.991; *p* < 0.01)
(B) Y = 0.637X − 0.011 (n = 5; r = 0.997; *p* < 0.001)
(C) Y = 0.659X + 0.038 (n = 5; r = 0.947; *p* < 0.02)

It should be emphasized that the data of Vallejo et al. [13] were used with the consent of Dr. Ursula Rinas and that the equation of this curve was calculated by us based on the published data.

### 3.3. Fermentation of hBMP-2 in a Bioreactor

**Figure 7:**
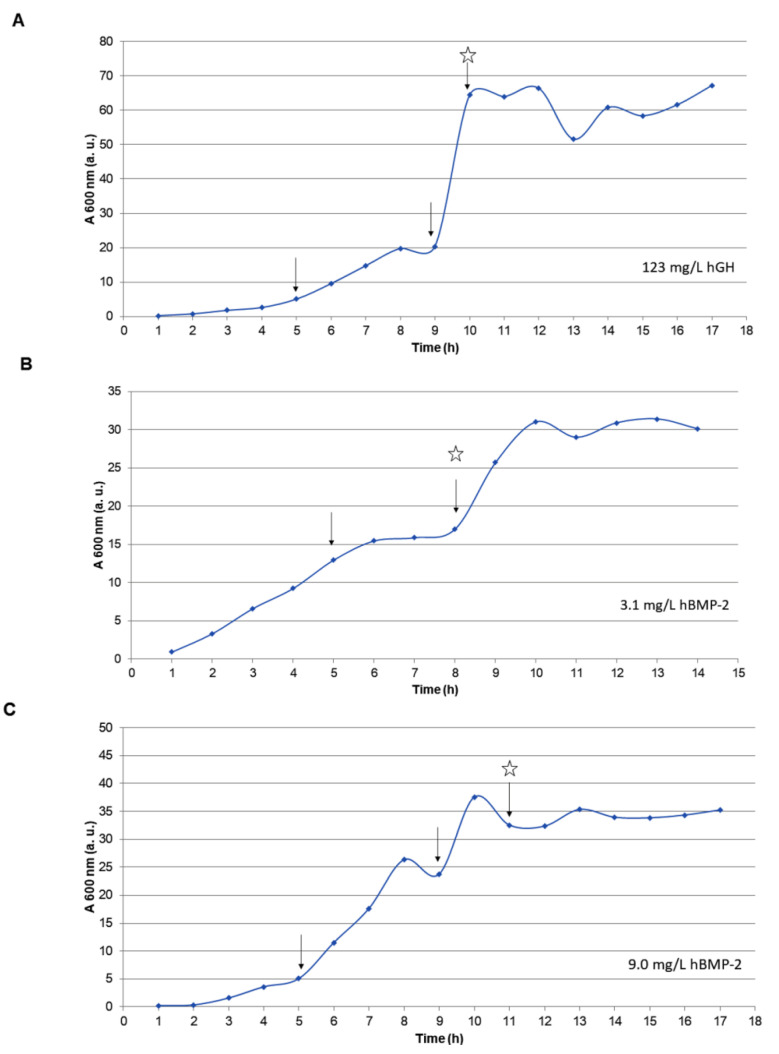
Bioreactor production with 5 L of broth. (**A**) W3110-λ_PL_-hGH-Rep/42 °C with Jensen 4× broth, (**B**) W3110-λ_PL_-hBMP-2-Rep/42 °C with Jensen 2× broth, and (**C**) W3110-λ_PL_-hBMP-2-Rep/42 °C with Jensen 4× broth. The arrows indicate the addition of glucose (fed-batch) at A_600_ ≥ 5 units, and the star an increase of temperature from 30 °C to 42 °C.

The specific and volumetric expression provided by the λ_PL_-DsbA-BMP-2 vector in the W3110 strain + repressor, in Erlenmeyer, were 0.16 ± 0.05 μg/mL/A_600_ and 0.71 ± 0.37 μg/mL (n = 4), respectively (Table 2). The same fermentation was then carried out in a 20 L bioreactor, under controlled conditions of pH, stirring, and dissolved oxygen level, using rich medium and glucose addition at an initial volume of 5 L, as described by Jensen and Carlsen [33]. Figure 7 shows a comparison with a repetition of an optimal experiment carried out by our research group for bioreactor production of hGH [30]. In the previous work, the hGH volumetric expression was 94.8 mg/L, while the same strain, under the same conditions, provided an even higher value, i.e., 123 mg/L, in the current work. The best performance for our hBMP-2, obtained with Jensen 4× broth, was only 9 mg/L, pointing to the necessity of additional investigation and process optimization.

### 3.4. In Vivo Bioassay Based on Rat Calvarias Critical-Size Defect Treatment

**Figure 8:**
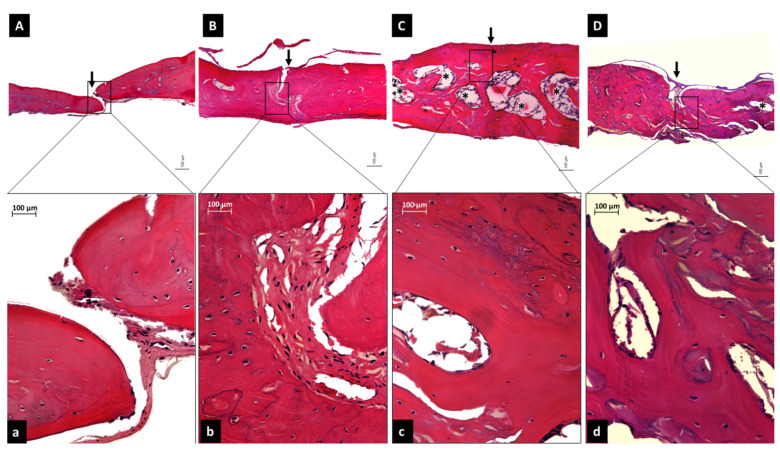
Histologic aspect of the central area of bone defects in all groups at 60 days (hematoxylin and eosin staining). The negative control, NC group (**A**), and absorbable collagen sponge group, ACS, (**B**) showed non-closure of the bone defect (black arrow) and remaining connective tissue, while the Infuse^®^ (**C**) and hBMP-2 Biosintesis groups (**D**) showed the closure of the defects (black arrow) and bigger vessels in the defect area (*); moreover, peripheral connective tissue was noticed in the hBMP-2 Biosintesis group. Higher magnification (**a**–**d**) of the areas within the black frames show the amount of newly formed tissue. NC (**a**) and ACS (**b**) showed a gap filled with connective tissue while (**c**,**d**) showed a significant formation of bone, with more bone for the Infuse^®^ group (**c**). Scale bars: 100 µm.

The histological analysis showed a complete calvaria defect closure at 60 days for the Infuse^®^ (INF) and hBMP-2 Biosintesis groups. The major difference between the groups regarded the bone volume and the blood vessels (Figure 8). The negative control (NC) group showed the thinnest bone height, with few blood vessels in the Newly formed Bone Area (NBA). In the absorbable collagen sponge, the ACS group, a slight increase in bone height and a small amount of blood vessels were noted, but some connective tissue remained in the central area, and there were ACS fragments on the edge of the newly formed bone.

The hBMP-2 groups (Infuse^®^ and hBMP-2 Biosintesis) showed almost twice as much bone height as the control group. Large vessels were observed in the central area of both groups, with a larger newly formed bone area.

**Figure 9:**
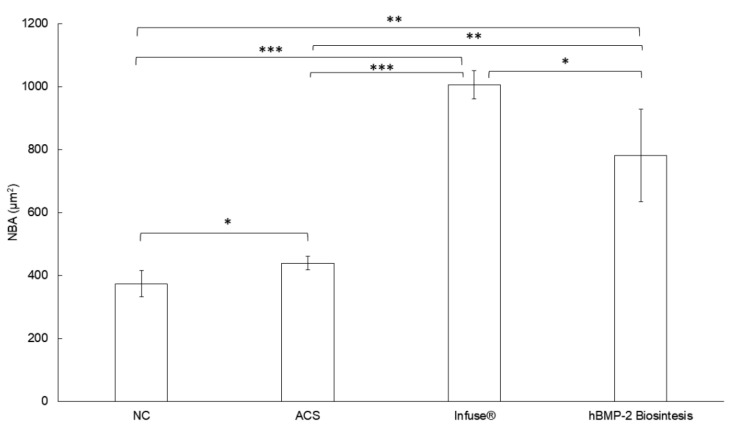
Histometric data for Newly formed Bone Area (NBA), 60 days after surgery, for the four groups analyzed. * *p* < 0.05; ** *p* < 0.005; *** *p* < 0.001.

The histometric analysis for newly formed bone area (NBA) showed 1,006 ± 45 µm^2^ for the Infuse^®^ group, 781 ± 147 µm^2^ for the hBMP-2 Biosintesis group, 439 ± 22 µm^2^ for the ACS group, and 374 ± 42 µm^2^ for the NC group (Figure 9). Concerning NBA, statistical analysis showed that the two hBMP-2 groups were significantly different (*p* < 0.005 or *p* < 0.001) in comparison with the two control groups. Our preparation did, however, exhibit a slightly lower osteoinduction compared to Infuse^®^ (*p* = 0.027).

## 4. Discussion

hBMP-2, one of the most efficient osteoinductors ever described, has now been successfully synthesized in *E. coli* periplasm in its natural, correctly folded, and authentic form, without the initial methionine, typical of cytoplasmic products, that frequently induces an undesired immunological reaction. For this purpose, an original expression vector based on hBMP-2 cDNA, the λ_PL_ promoter, the DsbA signal sequence, and the Amp^R^ gene was constructed and used to transform the *E. coli* W3110 strain. The identity of hBMP-2 was confirmed via Western blotting, RP-HPLC, and HPSEC by comparison with the cytoplasmic dimeric form of met-hBMP-2 from GenScript. Given that the bioactive form of this protein is the dimer, our efforts were directed to its purification through three steps, a HiTrap heparin affinity chromatography and two HPSECs, with the final product showing >95% purity.

On the other hand, the molar masses of the most intense periplasmic structures showing immunologic activity (Figure 2B) are around 35–38 kDa and 60–80 kDa. This deserves further investigation considering that, as pointed out by Israel et al. (1992), CHO-derived hBMP-2 contains a predominant bioactive protein of 30kDa. Of course, CHO cells are known to glycosylate proteins, while normal *E. coli* cells do not. In this context, we confirmed that some of these homodimeric forms are probably variants that differ only as a result of proteolytic processing at their amino termini [10,31]. Indeed, in MALDI–TOF-MS, our hBMP-2 exhibited a constant peak of ~31 kDa (results not shown). This may be due to the fact that the periplasmic bands are formed naturally, while the cytoplasmic hBMP-2 bands are constructed in order to obtain the homodimer. As stated by Quaas et al. [8], the intricate disulfide-bond pattern of the complex cysteine-knot scaffold was not present in the inclusion bodies with embedded disulfide-linked hBMP-2 but was formed later during classical refolding of the reduced protein under appropriate redox conditions. Therefore, it would not be a surprise to find that some of these high-molecular-weight periplasmic structures have good biological activity.

As a preliminary bioactivity determination, the classical in vitro assay based on induced alkaline phosphatase activity in murine myoblastic C2C12 cells was applied to our product, using the GenScript preparation for comparison. The slopes of the two dose–response curves, considered to be an indication of relative potency, were very similar: 0.637 A_405_/μg and 0.697 A_405_/μg for the GenScript preparation and for our product, respectively. These potencies were, moreover, not so different from that derived from an analogous renatured and purified cytoplasmic hBMP-2 (0.533 A_405_/μg), reported by Vallejo et al. [13] and already mentioned in previous work [31]. It is particularly noteworthy that, after two purification steps, the specific activity of our product was already very close to that of the GenScript preparation.

The use of an in vivo biological assay based on the osteoinductive potential of hBMP-2 for the treatment of calvarial critical-size defects in rats is, in our opinion, one of the most valuable aspects of our work. This assay, derived from a recent publication of Nakamura et al. [35], is widely considered to be a type of “gold standard” for testing the bone regeneration capacity of osteoinductors. Numerous pre-clinical studies have been conducted using different carriers in a variety of animal models, as described in a recent and extensive review [36]. The assay is particularly relevant because it employs two critical-size defects produced in the same animal, each one 5 mm in diameter, distant from each other by at least 2 mm: one defect serves as the standard, while the other evaluates the efficacy of the product under test. Critical size means that the defect cannot be closed spontaneously by new bone tissue unless a bone generation procedure is performed. The calvarial model is thus considered to be highly efficient for evaluating bone regeneration, due to its convenience, reproducibility, and low invasiveness, as well as the fact that it is a bone area that is not subjected to any strenuous activity [37,38,39,40]. In this context, it should be noted, that most studies that synthesized hBMP-2 in bacteria relied primarily on the alkaline phosphatase in vitro assay rather than evaluating the in vivo bioactivity of their preparation [8,13,20,22,32,41].

Our product was evaluated by comparison with CHO-derived Infuse^®^, which can be considered an international reference preparation for clinical use, with a collagen sponge control, and with a negative control. Statistically, both hBMP-2 preparations were significantly different from the two controls, and hBMP-2 Biosintesis presented a slightly lower potential in comparison with Infuse^®^. While literature data [24,25,26,27] report a comparable clinical efficacy of *E. coli*-derived versus glycosylated CHO-derived hBMP-2, in our hands these products showed different potencies, not only in the in vivo bioassay but also in the in vitro bioassay in C2C12 cells, as described in previous work [31]. Prohibitive prices limited our analyses to only two commercially available products: CHO-derived Infuse^®^ and cytoplasmic met-hBMP-2 from GenScript.

The experience of our research group in producing *E. coli*-derived recombinant proteins allowed us to expeditiously run a preliminary large-scale fermentation process under controlled bioreactor conditions, with the W3110 strain transformed with the pλ_PL_-DsbA-BMP-2 expression vector. The model we followed was the one previously employed for hGH production, starting from a similar vector construction and the same bacterial strain and fermentation process: pλ_PL_-DsbA-hGH, in the W3110 strain harboring the transcription repressor gene cIts. The fed-batch fermentation, with glucose addition and activation at 42 °C, based on the pλ_PL_-DsbA-BMP-2 expression vector, unfortunately provided a low yield, pointing to the need for further studies and improvement. Nonetheless, with a 150 L bioreactor, our laboratory has the potential to obtain ~1.35 g of hBMP-2 in just a single fermentation process, which is relatively satisfactory, considering that this protein is an extremely high added-value product used clinically at the microgram level.

## Figures and Tables

**Table 1:** Purity and potency increase during hBMP-2 purification.

Purification Step	Total Protein * (µg/mL)	hBMP-2 (µg/mL) **	Mass Fraction (%)	ALP Activity *** (A_405_)	ALP Specific Activity (A_405_/µg/mL)
Periplasmic fluid	2196	0.03	0.001	1.149	0.5 × 10^−3^
Heparin affinity	220	14.13	6.42	2.792	12.7 × 10^−3^
HPSEC	1.6	1.29	80.62	1.006	629 × 10^−3^
GenScript hBMP-2	5.00	3.70	74.00	3.2	640 × 10^−3^

* Determined by BCA; ** Determined by HPSEC; *** Determined by C2C12 assay.

**Table 2:** Specific and volumetric expression of hBMP-2.

Specific Expression (µg/mL/A_600_)	Specific Expression MEAN ± SD	Final Biomass (A_600_)	Final Biomass MEAN ± SD	Volumetric Expression (mg/L)	Volumetric Expression MEAN ± SD
0.20		4.46		0.89	
0.16		2.68		0.42	
0.19	0.16 ± 0.05	6.08	4.25 ± 1.43	1.14	0.71 ± 0.37
0.10		3.76		0.37	

## Data Availability

The data presented in this study are available on request from the corresponding author.
